# Ameloblastic fibrosarcoma of the mandible: A case report and mini review

**DOI:** 10.3892/etm.2014.1940

**Published:** 2014-08-29

**Authors:** YUAN-YUAN HU, MO-HONG DENG, LING-LING YUAN, YU-MING NIU

**Affiliations:** 1 Department of Stomatology, Taihe Hospital, Hubei University of Medicine, Shiyan, Hubei 442000, P.R. China; 2 Department of Oral and Maxillofacial Surgery, School and Hospital of Stomatology, Wuhan University, Wuhan, Hubei 430079, P.R. China; 3 Department of Pathology, Taihe Hospital, Hubei University of Medicine, Shiyan, Hubei 442000, P.R. China

**Keywords:** ameloblastic fibrosarcoma, ameloblastic fibroma, case report

## Abstract

Ameloblastic fibrosarcoma (AFS) is a rare malignant odontogenic neoplasm of the jaw. AFS is characteristically composed of a benign odontogenic epithelium and a malignant mesenchymal component. The posterior region of the mandible is the predominantly occupied site. In the present report, a new case of AFS in a 22-year-old male that originated from ameloblastic fibroma was described. Histologically, the tumor showed biphasic components: Benign epithelium and a malignant mesenchymal component. Immunochemical findings revealed that the tumor cells were positive for cluster of differentiation (CD) 34, vimentin, Ki-67 and p53, but negative for smooth muscle actin, S-100, CD68 and desmin. The clinical presentation, radiographic appearances and treatment measures were additionally described and reviewed.

## Introduction

Ameloblastic fibrosarcoma (AFS) is a rare malignant odontogenic neoplasm, first described by Heath in 1887 (1). AFS is composed of a benign odontogenic epithelium and a malignant mesenchymal component (2). The disease is characterized by bone destruction and swelling with pain. To date, only 91 documented cases have been reported in the English literature. The majority of AFS cases are located in the mandible and most commonly involve the posterior region. It has previously been shown that AFS can be regarded as the malignant counterpart of ameloblastic fibroma (AF) (3); however, AFS can also originate *de novo* (4). In this case report, a new case of AFS arising from a pre-existing AF is described. Furthermore, an analysis of the radiographic appearances and immunochemical features, as well as a review of the relevant literature, are presented. The patient provided written informed consent for this study.

## Case report

**Figure 1:**
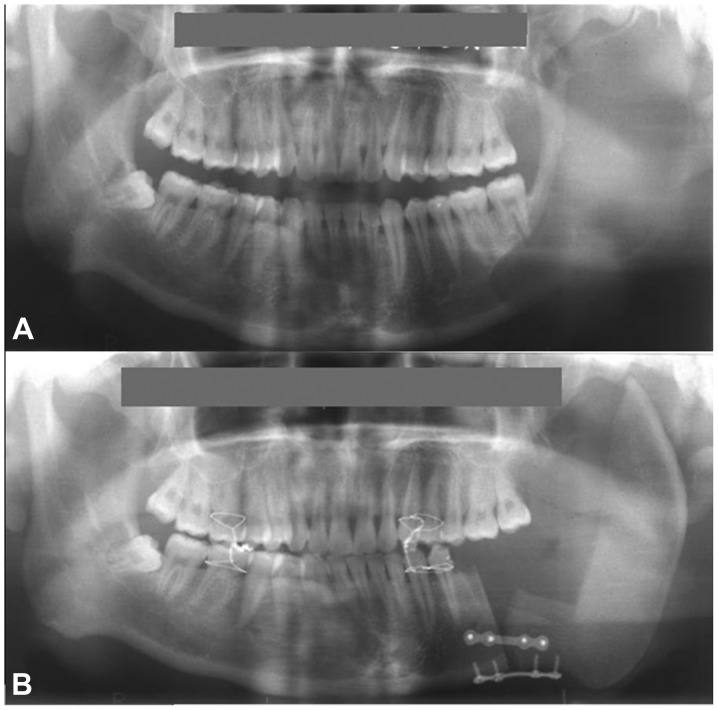
Panoramic radiographs showing (A) the unilocular radiolucent lesion of the left mandible and (B) reconstruction following the first surgery.

A 22-year-old Chinese male was referred to a local hospital in March 2005 with a two-month history of a painful swelling in his left mandible. Radiographic examination disclosed a unilocular radiolucent lesion located in the left mandible (Fig. 1A). An incisional biopsy was performed and the histopathological diagnosis was AF. The patient underwent left mandibulectomy and reconstruction with the ilia and iliac myocutaneous free flap (Fig. 1B).

**Figure 2:**
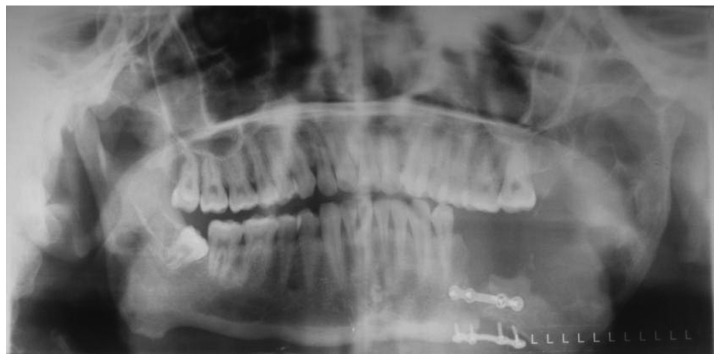
Panoramic radiograph showing an extensive, ill-defined, multilocular radiolucent lesion prior to the second surgery.

**Figure 3:**
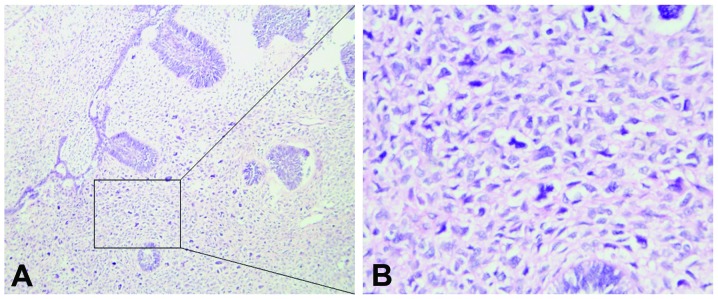
(A) Islands and nets of the odontogenic epithelium were embedded in the malignant mesenchymal component (HE staining; magnification, ×100). (B) The malignant mesenchymal component consisted of highly proliferative fibrous and spindle-shaped cells with pleomorphic, hyperchromatic and atypical figures (HE staining; magnification, ×400). HE, hematoxylin and eosin.

**Figure 4:**
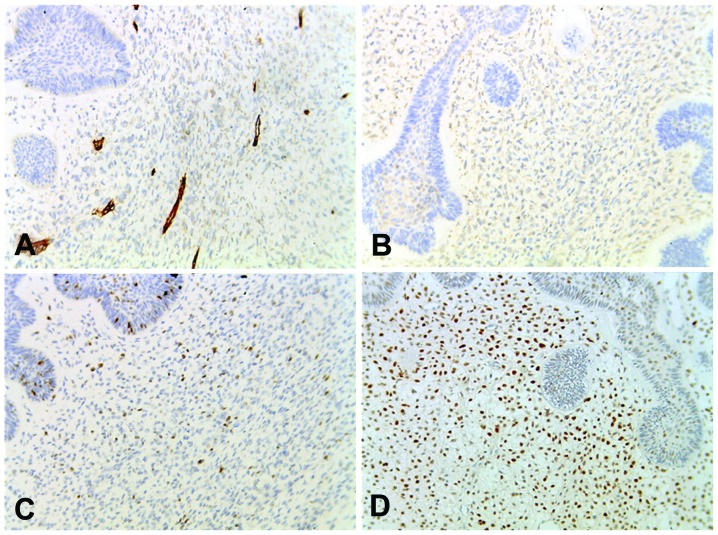
Immunohistochemistry (magnification, ×200) showed that the malignant mesenchymal tumor cells were positive for (A) cluster of differentiation 34 and (B) vimentin; the mesenchymal component was positive for (C) Ki-67, while (D) p53 protein was generally disclosed in the nucleus of the mesenchymal cells.

In September 2006, the patient came to our oral and maxillofacial center at the Department of Stomatology, Taihe Hospital (Hubei University of Medicine, Shiyan, China) and complained of the recurrence of the mass and acute pain within the last five months. Extraoral examination revealed a large, firm swelling extending from the premolar to the temporomandibular joint of the left mandible, as well as apparent asymmetry of the face. Intraoral examination showed a firm swelling located in the retromolar area of the left mandible, with ulceration of the mucosal membranes. The panoramic radiograph showed an extensive, ill-defined multilocular radiolucent area from the first premolar to the ramus of the left mandible (Fig. 2). The computed tomography (CT) scan revealed a 6.5×5.0 cm destructive lesion with bone resorption of the left mandible. An incisional biopsy was performed and the pathological diagnosis was consistent with AFS (Fig. 3). Immunohistochemically, the cells in the sarcomatous areas were positive for cluster of differentiation (CD) 34, vimentin, p53 protein and Ki-67, with a labeling index of ~22%, but negative for smooth muscle actin, S-100, CD68 and desmin (Fig. 4).

The patient underwent a wide left mandibulectomy including the surrounding soft tissue and destructive bone lesion. The final histopathological diagnosis was identical to that of the incisional biopsy. Postoperative radiotherapy with 50 Gy was implemented after the surgery for five weeks. The patient was under regular follow-up until 2011 (six years) after the surgery and showed no signs of recurrence.

## Discussion

AFS is a rare malignant neoplasm, consisting of two components: i) A reductive benign odontogenic epithelium arranged in an island and net pattern, dispersing in the mesenchymal tissue; and ii) malignant hypercellular mesenchymal tissue comprised of spindle and stellate cells, exhibiting nuclear pleomorphism and hyperchromatism (4,5). In the present case, immunohistochemical staining showed that the cells were predominantly positive for p53, Ki-67, CD34 and vimentin but negative for smooth muscle actin, S-100, CD68 and desmin in the mesenchymal tissue.

Radiographic examination is important to ascertain the correct diagnosis. Panoramic radiography can manifest a macroscopical, effective and low-cost image for the lesions involving the destructed bones. Furthermore, CT scans can provide more detailed information about the lesion and the association with the adjacent organs.

In this case, AFS was believed to show recurrence potential following conservative treatment. Compared with the initial lesions, recurrent lesions exhibit decreased epithelial components and increased pleomorphic and mitotic mesenchymal components (3,5,6). AFS is usually characterized by local invasiveness rather than regional or distant metastasis.

Previously, curettage, nucleation and local excision have typically been utilized in the surgical procedure (7). Since the proliferative potential and malignant degree of the mesenchymal component is elevated in the recurrent neoplasm, the eventual result is sarcomatous transformation (3,8). In this case, the inadequacy of the surgical boundary may have induced the recurrence of the tumor. Therefore, local control is strongly dependent on the extent of the initial resection, and conservative surgery must be abandoned and replaced by wide surgical resection with the surrounding soft tissue, particularly when the cortical plates have been perforated. Adjuvant postoperative radiotherapy and chemotherapy remain disputed. A number of cases involving chemotherapeutic medicines, such as cyclophosphamide and fluorouracil, have exhibited a positive result (9). Furthermore, the administration of radiotherapy (40–60 Gy) following surgery can produce effective results (4,10,11).

In conclusion, AFS is a rare malignant odontogenic tumor. It is difficult to discriminate AFS from AF when relying only on the histopathological results and clinical features. Resection with a wide margin is the optimal treatment strategy, and adjuvant chemotherapy and radiotherapy may additionally be used to reduce the recurrence rate and enhance the quality of life.
